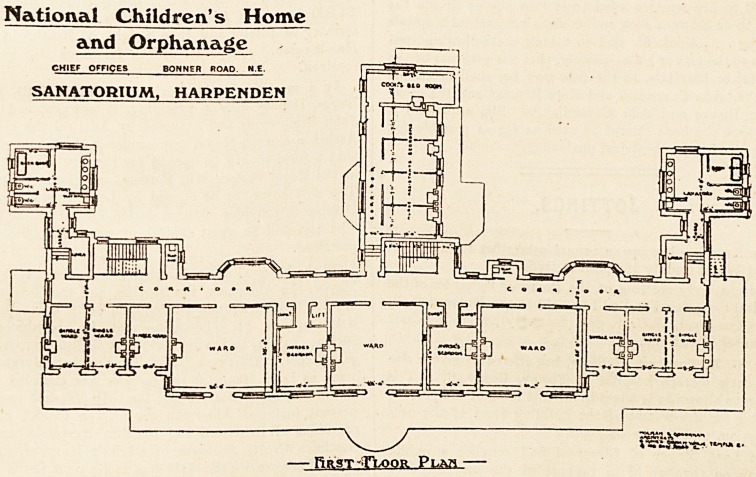# National Children's Home and Orphanage Sanatorium, Harpenden

**Published:** 1910-10-01

**Authors:** 


### NATIONAL CHILDREN'S HOME AND ORPHANAGE SANATORIUM, HARPENDEN.

THE building we illustrate to-day has been erected as a sanatorium for those inmates of the National Children's Home and Orphanage who are suffering from, or who show any tendency to, the various forms of tuberculosis.

The sanatorium occupies a site elevated some 430 feet above sea-level, facing nearly due south and commanding fine views of the surrounding country.

The plan has been arranged to take the fullest advantage of the south aspect, all the rooms occupied by patients being on the south front. In the centre of the ground floor is the dining-room, which has a large bay window at the south end, and French windows at the side leading out into the garden. On either side of the dining-room is a nurses' sitting-room; next to these are two wards, each for eight patients. The wards are well lighted with three large windows, the centre one of which is fitted with French casements giving access to a verandah. Adjoining each ward is a nurse's bedroom. At the extreme west end is a day-room for boys, and at the west end a waiting-room and surgery. At the north of these rooms a wide corridor runs the whole length of the building. Two large bay windows in this corridor afford cool retreats for patients in hot weather. At each end of the corridor the sanitary offices are placed in wings projecting to the north. These comprise cloak-rooms, lavatories, bathrooms and w.c.'s, and are efficiently isolated from the main corridor by cross-ventilated lobbies. Doors in the lobbies afford direct access from the grounds to the cloak-rooms and sanitary offices.

The central wing projecting to the north contains the kitchen offices, with heating chamber in basements.

The upper floor contains three wards for eight patients each, six single wards, and two nurses' bedrooms all on the south front. All these rooms are provided with French windows giving access to a wide balcony, so that all the patients can be wheeled into the open air in their beds. The sanitary offices are over those below, the space occupied by the cloak-rooms being on this floor utilised partly by the passage for access to the sanitary offices and partly by linen-rooms. The difference of level between the floor of the sanitary offices and the ward floor involves four steps. This seems an undesirable arrangement.

The kitchen wing contains bedrooms for the staff.

No provision appears to be made of bathrooms or w.c.s for nurses or servants—or if there has, there is no indication on the plan of it.

Though not clearly indicated on the plan there is an outside teak staircase at one end of the balcony which affords alternative means of escape in case of fire.

There is a detached laundry some sixty feet away from the main laundry.

The buildings were designed by Messrs. Holman and Goodrham, architects, of London.

— FIRST FLOOR PLAN —

## Figures and Tables

**Figure f1:**